# Genomic architecture of inflammatory bowel disease in five families with multiple affected individuals

**DOI:** 10.1038/hgv.2015.60

**Published:** 2016-01-07

**Authors:** Anna B Stittrich, Justin Ashworth, Mude Shi, Max Robinson, Denise Mauldin, Mary E Brunkow, Shameek Biswas, Jin-Man Kim, Ki-Sun Kwon, Jae U Jung, David Galas, Kyle Serikawa, Richard H Duerr, Stephen L Guthery, Jacques Peschon, Leroy Hood, Jared C Roach, Gustavo Glusman

**Affiliations:** 1 Institute for Systems Biology, Seattle, WA, USA; 2 Department of Molecular Microbiology and Immunology, Keck School of Medicine, University of Southern California, Los Angeles, CA, USA; 3 Novo Nordisk Research Center, Seattle, WA, USA; 4 Department of Pathology, Chungnam National University School of Medicine, Daejeon, Korea; 5 Aging Intervention Research Center, Korea Research Institute of Bioscience and Biotechnology (KRIBB), Daejeon, Korea; 6 Pacific Northwest Diabetes Research Institute, Seattle, WA, USA; 7 Luxembourg Centre for Systems Biomedicine, University of Luxembourg, Esch-sur-Alzette, Luxembourg; 8 Division of Gastroenterology, Hepatology and Nutrition, Department of Medicine, Clinical and Translational Science Institute, School of Medicine, University of Pittsburgh, Pittsburgh, PA, USA; 9 Department of Human Genetics, Graduate School of Public Health, University of Pittsburgh, Pittsburgh, PA, USA; 10 Division of Pediatric Gastroenterology, Hepatology and Nutrition, Department of Pediatrics, University of Utah, Salt Lake City, UT, USA

## Abstract

Currently, the best clinical predictor for inflammatory bowel disease (IBD) is family history. Over 163 sequence variants have been associated with IBD in genome-wide association studies, but they have weak effects and explain only a fraction of the observed heritability. It is expected that additional variants contribute to the genomic architecture of IBD, possibly including rare variants with effect sizes larger than the identified common variants. Here we applied a family study design and sequenced 38 individuals from five families, under the hypothesis that families with multiple IBD-affected individuals harbor one or more risk variants that (i) are shared among affected family members, (ii) are rare and (iii) have substantial effect on disease development. Our analysis revealed not only novel candidate risk variants but also high polygenic risk scores for common known risk variants in four out of the five families. Functional analysis of our top novel variant in the remaining family, a rare missense mutation in the ubiquitin ligase TRIM11, suggests that it leads to increased nuclear factor of kappa light chain enhancer in B-cells (NF-κB) signaling. We conclude that an accumulation of common weak-effect variants accounts for the high incidence of IBD in most, but not all families we analyzed and that a family study design can identify novel rare variants conferring risk for IBD with potentially large effect size, such as the TRIM11 p.H414Y mutation.

## Introduction

Inflammatory bowel disease (IBD, [MIM 266600]) is a chronic inflammatory condition of the gastrointestinal tract that affects about 3.7 million people in the US and Europe and shows increasing incidence in Asia.^1,2^ The two subtypes of IBD are Crohn’s disease (CD) and ulcerative colitis (UC). CD is characterized by discontinuous, transmural inflammation that can affect any part of the gastrointestinal tract, whereas UC presents with continuous inflammation that is usually restricted to the mucosa of the large intestine.

The strongest known risk factor for IBD is having an affected first-degree relative.^3^ Compared with the general population, siblings of CD patients are at 20- to 35-fold increased risk for developing CD, and siblings of UC patients are at 8- to 15-fold increased risk.^4^ Overall, a family history positive for IBD is reported by 5–16% of CD patients and 8–14% of UC patients.^4–6^ This implies a strong genetic contribution that is indeed demonstrated by the success of genome-wide association studies (GWAS). The most recent meta-analysis of CD and UC GWAS identified and validated 163 IBD-associated loci with genome-wide significance by combining data from a total of 75,000 cases and controls.^7^ Most of the identified loci confer risk to both subtypes.^7^

GWAS identify associations with common variants (usually >5% minor allele frequency), which have small effect sizes. Indeed, the highest odds ratio reported for a genetic variant influencing IBD is 3.99 for a loss-of-function variant in NOD2 (p.L1007fs).^8^ Carrying one copy of this risk allele quadruples the probability of developing CD from the population background prevalence of a quarter of a percent to 1%. To estimate the combined effect of multiple risk variants present in an individual's genome, a polygenic risk score can be calculated.^9^ Owing to the lack of knowledge about interactions between genetic variants and between genes and environment, polygenic risk score models typically assume independence among these factors. Accordingly, polygenic risk scores are calculated by multiplying the odds ratios of each risk allele that is present in a genome and taking the natural log of the product.^9^ The predictive value of the polygenic risk score, that is, the degree to which it explains the disease variance between affecteds and unaffecteds, depends on the genetic contribution to the disease and on the extent to which the variants that have a role have been identified. For IBD, taking all the 163 associated variants into account was reported to explain 13.6% of the disease variance for CD and 7.5% of the disease variance for UC.^7^ This is only a fraction of the 50% CD heritability and 19% UC heritability, respectively, that were observed in twin studies.^10^ We expect that additional genetic risk variants exist and that their identification will improve the predictive value of the polygenic risk score. Rare variants are not typically assessed by GWAS and may contribute to the ‘missing heritability’. We therefore analyzed five families with multiple (three to six) IBD-affected family members to test the hypothesis that low frequency variants (<3% allele frequency) are shared among affected family members and contribute to disease risk with intermediate to high effect sizes within these families.

## Materials and Methods

### Description of cohort

**Figure 1:**
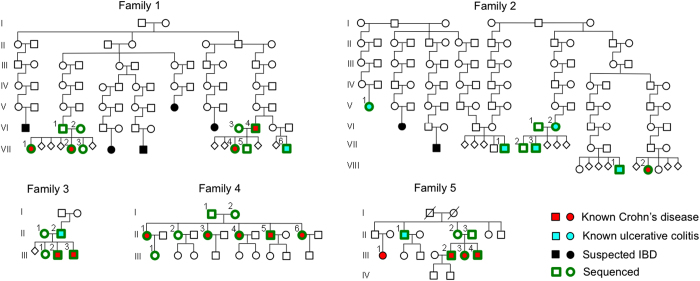
Pedigrees of the IBD-affected families. IBD, inflammatory bowel disease.

We studied five American families of European descent (Figure 1). Families 1–3 were recruited at the University of Utah with informed consent through study protocol #48821 approved by the IRB (Institutional Review Board). Families 4 and 5 were recruited through IRB-approved study protocol IRB0503185 at the University of Pittsburgh. Diagnosis of IBD was based on established criteria and required appropriate symptoms, exclusion of infections and objective evidence of inflammation on endoscopy, histology and/or radiology.^11^ An overview of disease onset and clinical features of the affected family members is provided in Supplementary Table 1.

### Whole-genome sequencing

DNA was extracted from blood samples. Paired-end library preparation,^12^ whole-genome sequencing (WGS), alignment to the reference genome (NCBI human genome assembly build 37) and variant calling were performed by Complete Genomics (Mountain View, CA, USA). The average coverage was greater than 40×.

### Identification of candidate variants

Each family was analyzed separately because we were expecting rare family-specific risk variants, possibly in different genes in each family. To identify candidate variants, we used the Ingenuity Variant Analysis software (Qiagen, Hilden, Germany) and the Family Genomics toolkit.^13^ We evaluated population frequencies of variants using Kaviar, a software program that estimates variant frequencies by taking into account a variety of public data sources as well as non-public genome sequences available from studies at the Institute for Systems Biology.^14^ The latest update of Kaviar includes genetic data from 77,238 individuals, comprising populations from around the world but with a bias for European-descent populations. We filtered for single-nucleotide variants and small indels that had Complete Genomics sequencing quality scores ⩾35, allele frequency ⩽3%, and a putative effect on gene function or activity, i.e., loss-of-function, missense or splice site mutations, or variants that putatively affect microRNA binding, promoter activity or enhancer activity. In addition, we filtered for variants that affect genes that are known to be of relevance for IBD in the Ingenuity Knowledge Base. These are (i) genes in a set of 292 genes directly connected to IBD, (ii) genes with interactions to any of these 292 genes or (iii) genes that have a connection with any of the 81 substances that have been related to IBD (e.g., IBD therapeutics).

### Computation of polygenic risk score

We divided a set of 163 previously identified IBD risk loci and their respective odds ratios^7^ into lists of 140 CD loci and 133 UC loci. One hundred and ten loci confer risk to both IBD subtypes and were included in both lists, but with IBD subtype-specific odds ratios. Because this set of loci does not contain two of the three well-characterized CD-associated loss-of-function variants in the *NOD2* gene, we added these loci and calculated odds ratios for all the three loci based on reported frequencies in cases and controls in different European populations.^15^ Specifically, we used the following odds ratios: p.R702W, 2.023; p.G908R, 3.500; p.L1007 fs; 4.255. To calculate the polygenic risk score, we took the natural log of the product of the odds ratios of each risk variant that is observed in an individual’s genome, i.e., if a risk variant is homozygous, it is multiplied twice; if it is heterozygous, it is multiplied once; and if it is absent, it does not contribute to the risk score. As controls we used 1,096 ISB in-house WGS from non-IBD family studies that were also sequenced by Complete Genomics. None of the genomes were derived from a cancer sample.

### Protein modeling

We modeled the functional impact of mutations in the PRY-SPRY domain on TRIM protein folding and stability with the Rosetta software.^16,17^ As no crystal structure for TRIM11 was available, we used the Protein Data Bank template structure of the PRY-SPRY domain of TRIM20 (Protein Data Bank ID 2WL1). For both wild-type and mutant proteins, we sampled conformations 100 times using a Monte Carlo procedure to minimize the estimated total free energy of protein folding. The change in free energy upon mutation (ΔΔG) estimates the impact of the mutation on folding and stability, with positive ΔΔG values indicating destabilization.

### Reporter assays

Wild-type TRIM11 was mutated to TRIM11 p.H414Y using a site-directed mutagenesis kit (Life Technologies, Carlsbad, CA, USA). HEK293T cells were transfected with reporter plasmid that harbors firefly luciferase under the control of (1) the promoter of nuclear factor of kappa light chain enhancer in B-cells (NF-κB1) or (2) the interferon signaling response element from the promoter of interferon-induced protein with tetratricopeptide repeats 1, in addition to TK-Renilla luciferase control plasmid, TANK-binding kinase-1 (TBK1)-expressing plasmid and wild-type or mutant TRIM11. At 24 h after transfection reporter luciferase activity was measured using the Dual-Luciferase Reporter Assay System (Promega, Madison, WI, USA).

### Immunostaining

Tissue samples from the intestine of a 5-month-old C57BL6 mouse were fixed in 10% buffered formalin and embedded in paraffin. Four-micrometer-thick sections from the paraffin blocks were used. After deparaffinization and antigen retrieval by pressure cooker in 10 mmol/l sodium citrate buffer (pH 6.0) at full power for 4 min, tissue sections were treated with 3% hydrogen peroxide for 10 min. The primary antibody for TRIM11 (1:100 dilution, rabbit polyclonal; Proteintech, Chicago, IL, USA) was incubated for 30 min followed by treatment with the EnVision-HRP reagent (Dako, Carpinteria, CA, USA) for 30 min. The slides were then sequentially incubated with DAB chromogen for 5 min, counterstained with Meyer’s hematoxylin and mounted for microscopy. All immunostaining steps were carried out at room temperature.

## Results

### Sequencing of five IBD families

We used WGS to analyze 38 individuals from five families including 15 CD patients, eight UC patients and 15 unaffected first-degree family members (Figure 1). Family 1 displays distant relationship over 10 generations between the two branches of the family that were analyzed. Four CD patients and one UC patient in the family were analyzed. Disease status is not known for all individuals in the older generations, but several additional family members are suspected to have (had) IBD. In Family 2, individual 2-V-1 is related to 2-VII-1 over 10 generations but not to other analyzed individuals of the family. In addition, three other branches of the family were analyzed that are distantly related to each other. Four family members have UC and one has CD. Family 3 is a nuclear family with an UC-affected father and two CD-affected children. Family 4 is a nuclear family with two unaffected parents but five of six children diagnosed with CD. Individual 4-III-1 was included in the study because she is the oldest from her generation and has reached an age at which the 4-II generation had already developed CD. Family 5 is a nuclear family with unaffected parents but three children diagnosed with CD. In addition, the UC-affected uncle of the children (5-II-1) was analyzed. Individual 5-III-1 was not available for analysis.

### Identification of risk variants

We first identified risk regions within which to focus our search for variants. Within family 1 and family 2, the affected family members are distantly related, i.e., separated by many meioses. Following our hypothesis that affected individuals in each family share risk variants, we aimed to identify genetic segments that are identical by descent among all affected family members. In pedigrees with distantly related affecteds, this approach greatly reduces the search space, and therefore the number of false-positive candidates, because with each meiosis the probability of sharing a segment that is not causing an ascertained phenotype, is approximately halved. According to Thomas *et al.,*^18^ and taking into account a recombination rate of 35.3 per meiosis,^19^ we calculated for families 1 and 2 the probability of observing an identical-by-descent segment shared by all affected family members (excluding individual 2-V-1 owing to different ancestry). The probabilities for shared segments are 0.07 and 0.01 in family 1 and 2, respectively, suggesting that no identical-by-descent regions are expected other than causative segments in these families. However, Inheritance State Consistency Analysis^13^ on the sequence data identified a 2.6 Mb (GRCh37 chr6: 80,111,322–82,671,825) and a 4.5 Mb (GRCh37 chr6: 43,216,439–47,754,537) shared segment in family 1, spanning several genes as well as a gene desert. In family 2, we did not identify a shared segment.

We next identified candidate risk variants using the filter criteria outlined in the Materials and Methods section. The 2.6 Mb shared segment in family 1 encompasses a single gene, Keratin 19, and did not have any candidate variants by our criteria, although these criteria are likely to filter some regulatory variants. We identified four candidate variants in the 4.5 Mb segment, potentially affecting *TJAP1, HSP90AB1, NFKBIE* and *RUNX2* (Table 1). By restricting the identity-by-descent analysis to CD patients, i.e., excluding 1-VII-6, we identified an additional candidate variant in *TRERF1*. We furthermore searched genome-wide in all the five families for candidate variants. The nuclear families 3, 4 and 5 as well as nuclear sub-branches of families 1 and 2 share many segments, so focusing on identity by descent represents much less of an advantage. Candidate variants are summarized in Table 1.

### Polygenic risk score analysis

**Figure 2:**
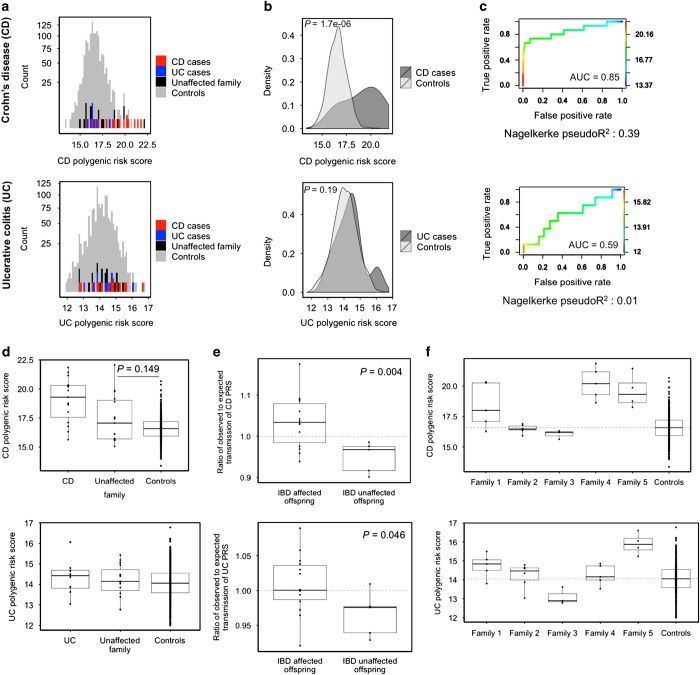
Polygenic risk scores. (**a**) Histograms of polygenic risk scores for CD (upper panel) and UC (lower panel) for all analyzed genomes (38 IBD family genomes and 1,096 control genomes). (**b**) Density plots of CD and UC polygenic risk scores for affected individuals versus controls (*P* values derived from one-sided Wilcoxon rank-sum test). (**c**) ROC for the discrimination between cases and controls using the polygenic risk scores, Nagelkerke pseudo *R*^2^ indicates explained variance by polygenic risk scores. (**d**) Comparison of CD (upper panel) and UC (lower panel) polygenic risk scores in familial IBD cases, unaffected family members, and controls (*P* value derived from one-sided Wilcoxon rank-sum test). (**e**) Ratio of transmission of CD and UC polygenic risk score from parents to affected offspring and unaffected offspring (dotted line indicates expected average transmission ratio of 1, *P* values derived from two-sided *t*-test). (**f**) Family-wise comparison of polygenic risk scores of affected family members versus controls (dotted line indicates average polygenic risk score in controls). AUC, area under the curve; CD, Crohn's disease; IBD, inflammatory bowel disease; ROC, receiver operator characteristic analysis; UC, ulcerative colitis.

In addition to the identification of new candidate risk variants, we analyzed the families for the burden of known single-nucleotide polymorphism associations with IBD. Using recent GWAS results on 163 genome-wide significant associations and the respective odds ratios,^7^ we calculated the UC and CD polygenic risk score for each individual (see Materials and Methods). We used 1,096 WGS controls available in-house at ISB from other family studies not related to IBD (Figure 2a). The CD polygenic risk scores of familial CD cases tend to be in the high range of scores compared with controls, with a significantly higher mean score (*P*=1.7e−06, Figure 2b). For familial UC cases, the mean UC polygenic risk score did not differ significantly between cases and controls (*P*=0.19). To estimate the predictive value of the polygenic risk scores, we used receiver operator characteristic analysis. The area under the curve was 0.85 for discriminating CD cases from controls using the CD polygenic risk score (Figure 2c). The UC polygenic risk score did not perform as well and resulted in an area under the curve of 0.59. To determine how much of the variance between familial cases and unrelated unaffected controls can be explained with the respective polygenic risk scores, we used logistic regression and calculated the Nagelkerke pseudo *R*^2^.^9^ The CD polygenic risk score accounted for 39% of the variance between familial CD cases and controls. The UC polygenic risk score accounted for just 1% of the variance between familial UC cases and controls.

The unaffected family members in our study have only a slightly increased CD polygenic risk score compared with controls (*P*=0.149); for UC, we do not detect a difference between unaffected family members and controls (Figure 2d). This may be owing to the small study size. If we assess only unaffected parents of CD-affected offspring, excluding any unaffected offspring, we see a stronger tendency towards higher risk scores for the unaffected parents compared with controls (*P*=0.03, data not shown). However, the transmission of risk variants from parents to offspring can deviate from the average 50% transmission rate per parent and this could lead to offspring with risk scores that exceed the parents' average risk score. We analyzed the transmission of risk scores from parents to offspring in our families and found that the ratio of observed risk score to expected risk score is higher in IBD-affected offspring than in unaffected offspring (Figure 2e).

Comparing the average risk score of affected individuals of each family revealed that family 3 has a CD and UC risk score below the average scores in controls. In contrast, in the other four families at least one of the two average risk scores is high (Figure 2f). Therefore, of the families we analyzed, family 3 seems most likely to carry additional genetic risk factors beyond the known variants (or haplotypes linked to them). Such genetic risk factors could include rare variants with intermediate-to-strong effects. The best candidate risk variant from our list of candidates for family 3 (Table 1) is a missense mutation in the ubiquitin E3 ligase TRIM11 [MIM 607868], c.1240G>A (p.H414Y). This variant is novel according to Kaviar.^14^ Members of the TRIM protein family have a role in host defense and regulate the NF-κB pathway,^20,21^ thus we hypothesized that TRIM11 could be involved in the IBD etiology. We used Sanger sequencing to validate the variant’s presence in the three affected family members and its absence in the unaffected parent and sibling. We evaluated the functional impact of the p.H414Y variant on the TRIM11 protein product by computational and experimental analysis.

### Analysis of the function of TRIM11 p.H414Y

**Figure 3:**
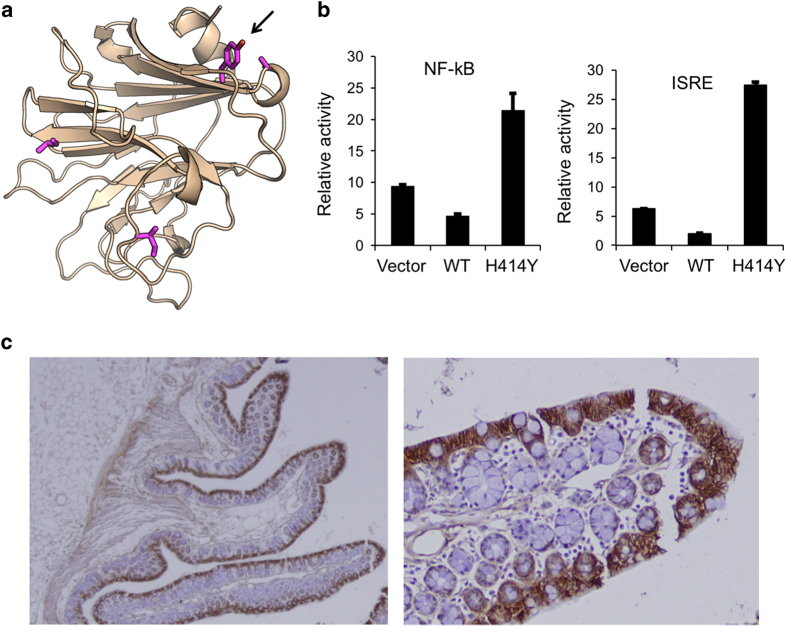
TRIM11 candidate variant p.H414Y. (**a**) Molecular model of the structure of TRIM11. The arrow points at the TRIM11 p.H414Y mutation. Other residues in sphere format indicate corresponding familial Mediterranean fever pathogenic mutations from TRIM20. (**b**) Relative activity in luciferase reporter assays testing the activity of the NF-κB promoter and the interferon signaling response element under ectopic expression of the empty vector (Vector), vector expressing wild-type TRIM11 (WT), and vector expressing p.H414Y mutated TRIM11 (H414Y). (**c**) Immunostaining of TRIM11 in murine intestinal sections, original magnification: left (×200), right (×400). ISRE, interferon signaling response element; NF-κB, nuclear factor of kappa light chain enhancer in B-cells.

The TRIM11 p.H414Y mutation maps to the PRY-SPRY domain, which is thought to mediate protein–protein interactions and which is shared by multiple members of the TRIM protein family.^21^ A better-studied member of the TRIM family is TRIM20 [MIM 608107], which causes the autoinflammatory disease familial Mediterranean fever (FMF) [MIM 134610].^22,23^ The most severe disease mutations, p.M680I, p.M694I, p.M694V and p.V726A, are located in the PRY-SPRY domain of TRIM20.^24^ We used protein modeling to estimate the impact of TRIM11 p.H414Y and the pathogenic TRIM20 mutations on folding and stability of the PRY-SPRY domain. Our results indicate that mutation to Y at the TRIM11 p.H414-corresponding position in TRIM20 is potentially destabilizing, although the effect is presumably weak with a ΔΔG of 4.22 kcal/mol. Values exceeding 3 kcal/mol suggest reduced stability,^25^ but many potential mutations in the PRY-SPRY domain have ΔΔG values of 50 kcal/mol or higher (Supplementary Figure 1). The TRIM20 pathogenic mutations have small negative ΔΔG values indicating no or a weakly stabilizing effect (Supplementary Table 2). Interestingly, all these mutations affect residues at the surface of the structure of the PRY-SPRY domain (Figure 3a). These findings suggest that, similar to the TRIM20 pathogenic mutations, TRIM11 p.H414Y may affect protein–protein interactions rather than protein folding and stability.

Residue p.414 of TRIM11 is moderately conserved among mammalian species, but poorly conserved among other TRIM family members. This suggests that this position may be important for the specificity of TRIM11 interactions with other proteins (Supplementary Figures 2 and 3).

We next used luciferase reporter assays to test the effect of the TRIM11 p.H414Y mutation experimentally. The assays were designed to report activity of the NF-κB1 promoter and activity of the interferon signaling response element from the promoter of interferon-induced protein with tetratricopeptide repeats 1. As shown in Figure 3b, expression of wild-type TRIM11 represses both NF-κB and interferon signaling; the mutant TRIM11 p.H414Y, however, has the opposite effect in that NF-κB and interferon signaling are both induced in response to its ectopic expression.

We further analyzed the location of TRIM11 expression using immunostaining of murine intestinal sections. TRIM11 localizes to the surface of intestinal villi and is specifically expressed in enterocytes and goblet cells (Figure 3c).

## Discussion

We analyzed whole-genome sequences of 38 individuals from five families with high incidence of IBD. In these families, we identified new rare candidate risk variants, and we found an increase in CD polygenic risk scores compared with controls. One family, however, did not show increased polygenic risks, suggesting the possible presence of a new rare IBD risk variant with substantial effect size. Therefore, we further characterized the function of the top candidate mutation in this family, TRIM11 p.H414Y. Our results from protein modeling, conservation analysis, functional testing and expression location indicate that TRIM11 p.H414Y is a plausible candidate for increasing the risk for IBD.

On the basis of the observed concordance rates in monozygotic twins, the heritability of CD was previously estimated to be up to 50%, and up to 19% for UC.^10,26,27^ In addition to the variability between studies, heritability estimates represent averages for which the underlying distributions are unknown; we are therefore cautious in interpreting heritability inferred from twin studies as leading to generalized insight into the genetic architectures of complex traits.^28^ If we, however, assume 50% CD heritability, then explaining 39% of the variance with the CD polygenic risk score suggests that 78% of the expected genetic contribution in our cohort is accounted for by the known variants. In other words, less than a quarter of the genetic risk in families 1, 2, 4 and 5 would be expected to be due to unknown, rare variants. It is nevertheless possible that the additional candidate variants we identified (but did not evaluate functionally) may have a role in IBD development in these families. For UC, the polygenic risk scores accounted for only a very small amount of the variance in these families (1%). One reason for this may be that we did not have as many UC patients as CD patients in our cohort and only one of the five families was mainly affected with UC. In general, the etiology of UC appears more complex than that of CD and may involve more contribution from non-genetic factors; the genetic associations with UC are usually smaller than for CD and the twin concordance rates are also much smaller. Therefore, explaining disease risk with a polygenic risk score based on known genetic associations is less feasible for UC than for CD.

The study by Jostins *et al.,*^7^ that identified the loci and odds ratios we used to compute the polygenic risk scores, reported that these loci explained 13.6% of the CD variance in their cohort. Our value of the explained variance for CD, 39%, is substantially higher. One possible reason for our elevated explained CD variance is that IBD patients from highly affected families may have higher polygenic risk than sporadic IBD cases (and the majority of IBD patients recruited for GWAS are sporadic cases). We suspect that the loci contributing to the high polygenic risk scores are the main driver for the high disease burden in the families we analyzed. The accumulation of the risk may be of a stochastic nature, in part driven by the increased risk scores in both parents and in part driven by the higher than average transmission rate of common risk variants from the parents to the affected children.

We identified TRIM11 p.H414Y as a risk candidate that appears to have a role in IBD susceptibility in family 3. TRIM proteins are members of the RING family of ubiquitin E3 ligases and have been implicated in the regulation of innate immune signaling, including the NF-κB pathway, in which TRIM-mediated ubiquitination has been shown at several steps in the pathway.^20,29^ Mutations in TRIM20, better known as MEFV, can cause the autoinflammatory disease FMF in a recessive or dominant fashion.^22,23^ Interestingly, IBD-like symptoms have been observed in FMF patients and TRIM20 mutations show association with IBD in populations with a high rate of TRIM20 mutations and FMF.^30–32^ The majority of the pathogenic FMF mutations are located in the PRY-SPRY domain of TRIM20, which is also the location of p.H414Y in TRIM11. Our protein modeling analyses suggest that, similar to known TRIM20 mutations, TRIM11 p.H414Y affects the protein surface structure rather than protein stability and may therefore impact protein–protein interactions. The function of TRIM11 is not well understood but recently TRIM11 was reported to inhibit the TANK-binding kinase-1 (TBK1) via protein–protein interaction that does not involve ubiquitination.^33^ TBK1 is part of the virus-sensing signaling cascade and induces NF-κB and interferon signaling.^34,35^ Our reporter assays showed the expected decrease in NF-κB and interferon signaling when wild-type TRIM11 was ectopically expressed. The observed induction of NF-κB and interferon signaling after ectopic expression of mutant TRIM11 p.H414Y could indicate a gain-of-function effect. It has been shown that TRIM11 does not directly inhibit the kinase activity of TBK1 so it is possible that instead the interaction of TBK1 with other activating or inhibiting molecules is altered by TRIM11 binding.^33^ We therefore hypothesize that the p.H414Y mutation might interfere with the inhibitory effect of TRIM11 on TBK1 and instead induces TBK1, leading to a stronger inflammatory signaling in response to viruses in the gut. Several components of the NF-κB signaling pathway have been associated with IBD before.^7^

We conclude from our results that the genomic architecture of familial IBD can involve both common and rare variant components. Common variants with small effect sizes, as have been identified by GWAS, appear to be sufficient to account for disease burden in many but not all families. In at least one family, we identified a rare variant plausibly contributing to IBD on the background of low polygenic risk. From our small study, we can neither confidently derive what fraction of families will fit each of the two models, nor what effect sizes rare variants may have in IBD. Large-effect rare variants may not be a typical driver for high incidence of IBD within families as (i) early genetic studies of IBD that used linkage analysis in families found, in comparison to monogenetic diseases, only modest linkage peaks, and no high-penetrance variants were identified in the linkage regions;^36^ (ii) deep resequencing of IBD GWAS loci did not reveal risk variants with odds ratios higher than 4.5;^8,37^ and (iii) rare large-effect variants are rather a hallmark of Mendelian diseases with early (childhood) onset^38,39^ whereas IBD has a late onset, usually in adolescence or early adulthood. The only context in which highly penetrant variants have been found to cause monogenic forms of IBD is in severe very-early-onset IBD; it has been suggested that, given the distinct genetic and phenotypic characteristics, very-early-onset IBD may, in fact, represent a new disease entity that is different from classical IBD.^40,41^ The families in this study do not fall into the very-early-onset IBD category.

To determine the effect sizes and disease risk contribution of rare variants such as the TRIM11 p.H414Y mutation, it will be necessary to study large cohorts of IBD cases and controls or families and use high-resolution approaches like WGS. We need more WGS studies of highly affected families to determine whether the results from our survey of five families will generalize to larger sets of families from many populations. In the future, we will not only need to include rare risk variants but also environmental factors in our risk models to predict disease risk with a level of accuracy that is clinically useful.

## Figures and Tables

**Table 1:** Candidate variants

*Chr*	*Position*	*Variant*	*Gene symbol*	*dbSNP ID*	*Gene region*	*Translational impact/ regulator*	*AF (%)*	*Zygosity*	*Family*	*Carrier*	*Presence in other families?*	*SIFT*	*PolyPhen-2*	*Distance to IBD*	*Method*
1	5923427	A>T	NPHP4	rs35641267	Exonic	Missense	0.1457	Comp-het (with rs17472401 and rs34248917)	5	**5-II-1**, 5-II-2, **5-III-2**, **5-III-3**, **5-III-4**	**2-V-1**, **2-VIII-1**	Damaging	Probably damaging	2 nodes	Genome-wide
1	5940243	G>A	NPHP4	rs17472401	Exonic	Missense	1.5121	Comp-het (with rs35641267)	5	5-II-3, **5-III-3**	**3-II-2**, 3-III-1, **3-III-2**	Damaging	Probably damaging	2 nodes	Genome-wide
1	5951013	C>T	NPHP4	rs34248917	Exonic	Missense	2.4400	Comp-het (with rs35641267)	5	5-II-3, **5-III-2**, **5-III-4**	No	Tolerated	Benign	2 nodes	Genome-wide
1	67631299	G>A	IL23R	rs187980208	Promoter	ETS1; SPIB; FOXC1	0.4564	Het	1	**1-VI-4**, **1-VII-4**	No	—	—	Direct	Genome-wide
1	67631416	A>G	IL23R	rs11465752	Promoter	NR4A2; Arnt::Ahr; GATA2; Pax2	0.3731	Het	1	**1-VII-6**	4-I-2, **4-II-3**, **4-II-6**	—	—	Direct	Genome-wide
1	228582573	G>A	TRIM11	—	Exonic	Missense	0	Het	3	**3-II-2**, **3-III-2**, **3-III-3**	No	Damaging	Benign	1 node	Genome-wide
3	47450362	C>T	PTPN23	rs761990455	Exonic	Missense	0.0026	Het	1	**1-VI-4**, **1-VII-4**, **1-VII-6**	No	—	Benign	1 node	Genome-wide
4	187455617	C>T	MTNR1A	rs762478338	Exonic	Stop-gain	0.0026	Het	3	**3-II-2**, **3-III-2**, **3-III-3**	No	—	—	1 node	Genome-wide
5	146258171	G>T	PPP2R2B	rs115018751	Exonic	Missense	1.7407	Het	1	**1-VI-4**, **1-VII-4**, **1-VII-6**	No	—	—	Direct	Genome-wide
5	146436501	A>T	PPP2R2B	rs149586822	Promoter	SOX10; Hltf	1.8915	Het	1	**1-VI-4**, **1-VII-4**, **1-VII-6**	No	—	—	Direct	Genome-wide
6	42236485	G>A	TRERF1	rs202210535	Exonic	Missense	0.0084	Het	1	1-VI-2, **1-VI-4**, **1-VII-1**, **1-VII-2**, 1-VII-3, **1-VII-4**	No	Damaging	Benign	1 node	Identical-by-descent
6	43473025	G>A	TJAP1	rs147994267	Exonic	Missense	0.4267	Het	1	1-VI-2, **1-VI-4**, **1-VII-1**, **1-VII-2**, 1-VII-3, **1-VII-4**, **1-VII-6**	No	Tolerated	Possibly damaging	1 node	Identical-by-descent
6	44214869	T>A	HSP90AB1	rs35074133	5'UTR	MEF2C; ELF1; E2F4; FOXA2; E2F6; WRNIP1; TBP; NFKB1; MXI1; TCF7L2; POLR2A; MAX; HSF1; JUND; CHD2; MYC; ZNF263; TAF1; YY1; FOXA1; HNF4G; ZNF143; STAT1; PPARGC1A; USF1; HDAC2; EP300; SP1; JUN; IRF1; TCF12	3.4775	Het	1	1-VI-2, **1-VI-4**, **1-VII-1**, **1-VII-2**, 1-VII-3, **1-VII-4**, **1-VII-6**	No	—	—	1 node	Identical-by-descent
6	44226433	C>T	NFKBIE	rs28385699	3'UTR	EBF1; TBP	0.5038	Het	1	1-VI-2, **1-VI-4**, **1-VII-1**, **1-VII-2**, 1-VII-3, **1-VII-4**, **1-VII-6**	5-II-2, **5-III-4**	—	—	1 node	Identical-by-descent
6	45295718	C>A	RUNX2	rs78404771	Promoter	MZF1_1-4	1.7832	Het	1	1-VI-2, **1-VI-4**, **1-VII-1**, **1-VII-2**, 1-VII-3, **1-VII-4**, **1-VII-6**	No	—	—	1 node	Identical-by-descent
6	52101833	C>T	IL17F	rs141798304	Exonic	Missense	0.0123	Het	1	**1-VI-4**, **1-VII-4**, **1-VII-6**	(yes)	Damaging	Benign	Direct	Genome-wide
6	91297992	T>G	MAP3K7	—	Promoter	MEF2A; TBP; FOXC1; FOXL1	0	Het	1	**1-VI-4**, **1-VII-4**, **1-VII-6**	No	—	—	Direct	Genome-wide
7	117267812	T>G	CFTR	rs34911792	Exonic	Missense	0.5174	Het	1	**1-VII-6**	No	Tolerated	Benign	Direct	Genome-wide
11	102248406	A>G	BIRC2	rs61754131	Exonic	Missense	0.1302	Het	5	**5-II-1**, 5-II-2, **5-III-2**, **5-III-3**, **5-III-4**	No	Damaging	Probably damaging	1 node	Genome-wide
12	101603524	C>A	SLC5A8	rs117927891	Exonic	Missense/ E2F1	1.1889	Comp-het? (with SLC5A10)	4	4-I-1, **4-II-1**, **4-II-3**, **4-II-4**, **4-II-5**, **4-II-6**	No	Tolerated	Benign	1 node	Genome-wide
12	129299599	G>A	SLC15A4	rs144816528	Exonic	Missense	0.1690	Hom	4	**4-II-1**, 4-II-2, **4-II-3**, **4-II-4 (**het in 4-I-1, 4-I-2)	No	Tolerated	Possibly damaging	2 nodes	Genome-wide
15	33381040	C>G	FMN1	rs34718785	Exonic	Missense	0.4403	Comp-het (with rs181008299, rs149624435)	5	5-II-3, **5-III-2**, **5-III-3**, **5-III-4**	No	—	—	2 nodes	Genome-wide
15	33381139	T>C	FMN1	rs181008299	Exonic	Missense	0.1658	Comp-het (with rs34718785)	5	5-II-2, **5-III-2**, **5-III-3**, **5-III-4**	No	—	—	2 nodes	Genome-wide
15	33445438	G>A	FMN1	rs149624435	Exonic	Missense	0.1820	Comp-het (with rs34718785)	5	5-II-2, **5-III-2**, **5-III-3**, **5-III-4**	No	Damaging	—	2 nodes	Genome-wide
16	16259596	G>A	ABCC6	rs41278174	Exonic	Missense	2.0327	Hom	5	**5-III-2**, **5-III-3**, **5-III-4** (het in 5-II-2, 5-II-3)	No	Damaging	Probably damaging	2 nodes	Genome-wide
17	18916758	C>T	SLC5A10	—	Exonic	Missense	0	Comp-het? (with SLC5A8)	4	4-I-2, **4-II-1**, **4-II-3**, **4-II-4**, **4-II-5**, **4-II-6**	No	Damaging	Possibly damaging	2 nodes	Genome-wide
18	30791889	C>G	CCDC178	rs117587736	Exonic	Missense	1.3178	Hom	5	**5-III-2**, **5-III-3**, **5-III-4** (het in 5-II-1, 5-II-2, 5-II-3)	No	Tolerated	Benign	2 nodes	Genome-wide
20	33674004	C>T	TRPC4AP	rs55993524	Intronic	Noncoding	8.5487	Hom	4	**4-II-1**, **4-II-3**, **4-II-4**, **4-II-5**, **4-II-6** (het in 4-I-1, 4-I-2)	**1-VII-6**	—	Benign	1 node	Genome-wide

‘Family’ indicates the family in which the variant was identified; ‘Carrier’ indicates carriers in these families with bold font indicating affected carriers; ‘Presence in other families?’ indicates if variant is present in one of the other IBD families studied; ‘SIFT’ indicates SIFT functional prediction;^42^ ‘Polyphen-2’ indicates Polyphen-2 functional prediction;^43^ ‘Distance to IBD’ indicates the number of nodes between the candidate gene and IBD according to the Ingenuity Knowledge Base; ‘Method’: indicates if the variant was identified in an identical-by-descent segment or by genome-wide analysis.

Abbreviations: AF, Kaviar allele frequency of the candidate variant; comp-het, compound-heterozygous; het, heterozygous; hom, homozygous; IBD, inflammatory bowel disease.
