# Supplementary material for: Genomic architecture of inflammatory bowel disease in five families with multiple affected individuals
Source: Hum Genome Var. 2016 Jan 7;3:15060–. doi: 10.1038/hgv.2015.60 (PMC4785573; doi:10.1038/hgv.2015.60)
Supplement: Supplemental Information [file hgv201560-s1.doc]

**Supplementary Figures and Tables**

**Supplementary Figure 1: Saturation scan of mutations in the TRIM20/2WL1 structure**

∆∆G energy scores of possible single nucleotide missense mutations for each position along the sequence. The pink arrow indicates the TRIM20 position p.S728 which corresponds to the TRIM11 p.H414 position. The pink dot represents the mutation to Y and purple dots represent other mutations at this position.

**Supplementary Figure 2: Alignment of the TRIM11 sequence across mammalian species**

BLASTP alignment of *Homo sapiens* TRIM11 to mammalian orthologs, focusing on the segment containing the candidate variant p.H414Y (purple).

**Supplementary Figure 3: Alignment of TRIM protein family members containing the PRY-SPRY domain**

BLASTP alignment of several *Homo sapiens* TRIM protein sequences. The position corresponding to H414 in TRIM11 is highlighted in purple.

**Supplementary Table 1:** Clinical features

| Family | Individual | IBD subtype | Age of onset | Clinical features and other remarks |
| --- | --- | --- | --- | --- |
| 1 | VI-4 | CD | ~30 | Colectomy, developed pyderma gangrenosum |
| 1 | VII-1 | CD | 18 | Fibrostenotic CD, required surgery |
| 1 | VII-2 | CD | 15 | Fibrostenotic CD, required surgery |
| 1 | VII-4 | CD | 16 | Inflammatory CD |
| 1 | VII-6 | UC | 12 | n/a |
| 2 | V-1 | UC | 15 | n/a |
| 2 | VI-2 | UC | childhood | n/a |
| 2 | VII-1 | UC | 11 | Colectomy for severe disease at age 16 |
| 2 | VII-3 | UC | 11 months | Severe disease |
| 2 | VIII-1 | UC | 11 | Colectomy for severe disease at age 13 |
| 2 | VIII-2 | CD | 16 | Moderate to severe disease |
| 3 | II-2 | UC | 45 | Transverse colon affected |
| 3 | III-2 | CD | 13 | Ileocolonic disease |
| 3 | III-3 | CD | 15 | Ileocolonic disease |
| 4 | II-1 | CD | 20 | Ileocolonic resection with temporary diverting stoma |
| 4 | II-3 | CD | 16 | n/a |
| 4 | II-4 | CD | 17 | Ileocolonic resections (three times) |
| 4 | II-5 | CD | 28 | Ileocolonic resections (two times) |
| 4 | II-6 | CD | 11 | Proctocolectomy with ileostomy due to rectal stricture |
| 5 | II-1 | UC | 48 | No active disease |
| 5 | III-2 | CD | 12 | Ileocolonic resection due to fibrostenosing CD |
| 5 | III-3 | CD | 23 | Prior ileal stenosis resolved by drug therapy |
| 5 | III-4 | CD | 12 | Ileocolonic resection due to terminal ileum stricture |

CD: Crohn's disease, UC: ulcerative colitis

**Supplementary Table 2:** Modeling of energetic consequences of TRIM20 mutations

| Position | Wild type | Mutation | ∆∆G [kcal/mol] |
| --- | --- | --- | --- |
| 680 | M | I | -2.259 |
| 694 | M | I | -1.19 |
| 694 | M | V | -1.691 |
| 726 | V | A | -1.347 |
| 728 | S | Y | 4.22 |
